# Low Skeletal Muscle Mass and the Incidence of Delirium in Hospitalized Older Patients: A Systematic Review and Meta-Analysis of Observational Studies

**DOI:** 10.1155/2023/4098212

**Published:** 2023-05-05

**Authors:** Yuhou Shen, Qianyi Wan, Rui Zhao, Yi Chen, Lin Xia, Yutao Wu, Shuomeng Xiao, Yong Wang, Lihao Zhao, Tao Li, Xiaoting Wu

**Affiliations:** ^1^Division of Gastrointestinal Surgery, Department of General Surgery, West China Hospital, Sichuan University, Chengdu 610041, China; ^2^Department of Oral and Maxillofacial Surgery, West China Hospital of Stomatology, Sichuan University, Chengdu 610041, China; ^3^Laboratory of Mitochondria and Metabolism, Department of Anesthesiology, National Clinical Research Center for Geriatrics, West China Hospital, Sichuan University, Chengdu 610041, China

## Abstract

**Background:**

Both low skeletal muscle mass and delirium are prevalent in older hospitalized patients, while their associations are unclear. This systematic review and meta-analysis aim to investigate the associations between low skeletal muscle mass and the incidence of delirium in hospitalized patients.

**Methods:**

The PubMed, Web of Science, and Embase were searched for relevant studies published before May 2022, and we conducted this systematic review and meta-analysis according to the PRISMA and MOOSE guidelines. The summary odds ratios (OR) and 95% confidence intervals (CI) were estimated, and subgroup analyses were also conducted according to the age and major surgeries.

**Results:**

Finally, nine studies with 3 828 patients were included. The pooled result showed no significant association between low skeletal muscle mass and the incidence of delirium (OR 1.69, 95% CI 0.85 to 2.52). However, sensitivity analysis suggested that one study caused a significant alteration of the summary result, and the meta-analysis of the remaining 8 studies showed that low skeletal muscle mass was significantly associated with an 88% increased incidence of delirium (OR 1.88, 95% CI 1.43 to 2.33). Furthermore, subgroup analyses indicated that low skeletal muscle mass was associated with a higher incidence of delirium in patients ≥75 years old or undergoing major surgeries instead of those <75 years old or without surgeries, respectively.

**Conclusions:**

Hospitalized patients with low skeletal muscle mass might have higher incidence of delirium, particularly in those of older age and undergoing major surgeries. Therefore, great attention should be paid to these patients.

## 1. Introduction

Sarcopenia is a progressive and generalized skeletal muscle disease that is characterized by the loss of skeletal muscle mass and function [1, 2], and low skeletal muscle mass is the most important diagnostic criterion of sarcopenia [3]. Old age is an important risk factor of low skeletal muscle mass. It is reported that after 50 years old, people could lose an average of about 15% of their skeletal muscle mass per decade [4], leading to an increased prevalence of low skeletal muscle mass in older populations [5]. Besides, low skeletal muscle mass is more prevalent in patients with cancer, with a median incidence of 43% as reported in a recent systematic review of 156 studies [6]. The normal skeletal muscle mass is important for health, and our previous study indicated that computed tomography (CT) or bioimpedance analysis (BIA) determined low skeletal muscle mass could lead to increased risks of many adverse health-related outcomes such as poor survival and increased postoperative complications [7].

Delirium, an acute state of brain failure, which is defined as an acute and fluctuating impairment in attention, awareness, and cognition [8–10]. A recent meta-analysis of 33 studies estimates that the overall prevalence of delirium is about 23% in medical inpatients [11]. While for patients undergoing high-risk surgeries such as hip fracture repair and cardiac surgery, the rate of postoperative delirium could be much higher [9, 10]. Delirium is significantly associated with poor outcomes in hospitalized patients, such as an increased risk for death, nosocomial complications, prolonged length of stay, and poor cognitive recovery [9]. Therefore, it is important to discover the delirium-related risk factors and prevent delirium for hospitalized patients.

Skeletal muscle, one of the largest metabolic organs, is thought to influence cognitive functions [12]. However, the associations between low skeletal muscle mass and the incidence of delirium are not well understood yet. Although some observational studies have looked at this issue recently, it is controversial whether skeletal muscle mass is significantly associated with the incidence of delirium in hospitalized patients [13–16]. The inconsistences of these studies might be caused by confounding factors such as the age and different disease conditions of included patients. Therefore, to better understand this issue, we conducted this systematic review and meta-analysis, which could be helpful to understand the relationship between skeletal muscle mass and delirium and prevent the delirium in hospitalized patients.

## 2. Methods

This systematic review and meta-analysis were registered at PROSPERO (CRD42022330979).

### 2.1. Study Search and Inclusion Criteria

We conducted and reported this systematic review and meta-analysis according to the PRISMA and MOOSE guidelines [17–19], and the PRISMA and MOOSE checklists were reported in the supplementary materials (available here). The PubMed, Web of Science, and Embase were searched independently by two authors for relevant studies about the associations between low skeletal muscle mass and delirium published before May 2022. The terms for searching were as follows: (“sarcopenia” OR “skeletal muscle loss” OR “low skeletal muscle” OR “muscle wasting” OR “muscle atrophy”) AND (“delirium” OR “deliration” OR “cognitive”). Detailed search strategies for each database were shown in the supplementary materials. Furthermore, the references of related studies were also reviewed for potential articles missed in the initial search. When the full-text studies are not available, we will contact the authors using e-mail.

All studies retrieved from the electronic databases were reviewed by two authors independently, and the disagreements were resolved by discussion. The inclusion criteria were: (1) observational studies about the hospitalized patients; (2) the skeletal muscle mass of each patient was assessed by CT, BIA or other methods; (3) the relevant data about the associations between skeletal muscle mass and delirium were reported; and (4) the studies were published in English. editorials, reviews, abstract, and animal studies were excluded from this systematic review and meta-analysis. For each included study, the quality was evaluated based on the Newcastle-Ottawa-Scale (NOS) [20].

### 2.2. Data Extraction

For each included study, the data were extracted by two authors independently, and the extracted data were as follows: the name of the first author and the year of publication; the country where the study was conducted; the design of the study; the assessment of skeletal muscle mass and the definition of low skeletal muscle mass; the assessment of delirium; the study population and number of patients; and the incidence of delirium or the estimates of effect size with 95% confidence intervals (CI) about the associations between skeletal muscle mass and the incidence of delirium.

### 2.3. Statistical Analysis

In this systematic review and meta-analysis, the software STATA version 15.0 was used for all statistical analyses. Considering the assessment of skeletal muscle mass and the definition of low skeletal muscle mass were inconsistent among the included studies, all the summary odds ratios (OR) and 95% CI were estimated with a random-effects model. The heterogeneity was assessed by *Q* statistic [21], and a *P* value of the *Q* test <0.1 or the *I*^2^ value ≥50% meant a statistically significant heterogeneity. The stability of the summary result was evaluated by sensitivity analysis, which was performed by omitting one study at a time during repeated analyses. If the removal of a study leads to a significant bias in the summary effect size and 95% CI, the study will be eliminated [22, 23]. The publication bias was visualized by funnel plots and assessed by Egger's test, and a *P* value of less than 0.1 was considered statistically significant publication bias. When publication bias existed, the trim-and-fill method would be used to estimate an adjusted summary OR and 95% CI and eliminate publication bias [23]. Furthermore, we also conducted subgroup analyses according to the age and whether patients undergoing surgeries. All statistical tests were two-sided.

## 3. Results

**Figure 1:**
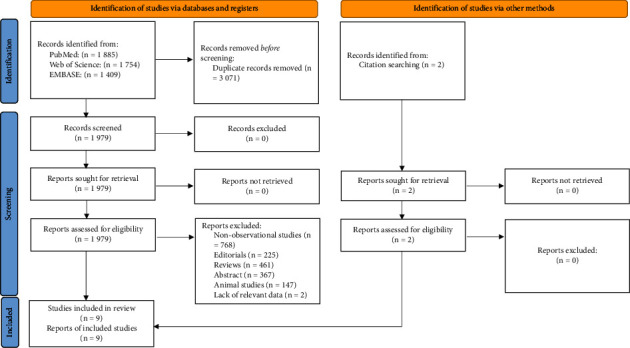
Flow chart of the study selection process.

From the three electronic databases and references of related studies, we identified 1,979 studies. After reviewing the title, abstract, and full text, nine studies [13–16, 24–28] were finally included in this systematic review and meta-analysis (Figure 1). The median NOS quality score was 8 (range from 6 to 8) (Table 1).

These 9 studies contained a total of 3,828 patients from America, Italy, Japan, South Korea, and the Netherlands. Only one study included patients with a mean age of 59.4 years old, and the other studies included patients with a mean or median age of over 60 years old. The skeletal muscle mass was assessed by CT or MRI in 6 studies and by BIA in 2 studies, and only one study assessed the muscle mass according to the calf circumference. Besides, patients in 7 studies underwent kinds of major surgeries. The detailed information of included studies was shown in Table 1.

**Figure 2:**
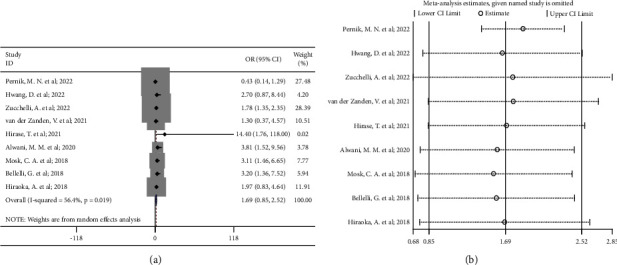
(a) Forest plot of the associations between low skeletal muscle mass and the incidence of delirium (all nine included studies); (b) sensitivity analysis of the nine included studies.

For the associations between low skeletal muscle mass and the incidence of delirium, the pooled result of 9 included studies showed no significant association existed (OR 1.69, 95% CI 0.85 to 2.52; *P*_heterogeneity_=0.02) (Figure 2(a)). However, sensitivity analysis suggested that only after removing the study by Pernik et al. [15], the pooled result turned to be significant (Figure 2(b)). Considering that in the study by Pernik et al. patients with higher skeletal muscle mass had a significantly higher BMI than those with low skeletal muscle mass (BMI 29.6 vs. 27.5, *P*=0.02), and a higher BMI could be associated with an increased risk of postoperative delirium [29–31]. To decrease the risk of bias, we removed the study by Pernik et al. in the subsequent analyses.

**Figure 3:**
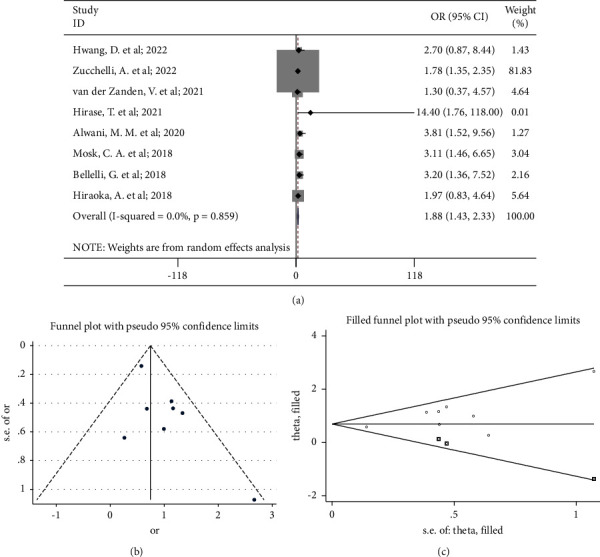
(a) Forest plot of the associations between low skeletal muscle mass and the incidence of delirium (eight studies after sensitivity analysis); (b) funnel plot of the publication bias; (c) plot of the trim-and-fill method.

The pooled result of the remaining 8 studies showed that low skeletal muscle mass was significantly associated with an 88% increased incidence of delirium in hospitalized patients (OR 1.88, 95% CI 1.43 to 2.33; *P*_heterogeneity_=0.86) (Figure 3(a)). However, the funnel plot and Egger's test suggested existence of publication bias (*P*=0.05) (Figure 3(b)). Therefore, the trim-and-fill method was conducted, and the adjusted summary result also showed that low skeletal muscle mass was significantly associated with an increased incidence of delirium (OR 2.00, 95% CI 1.42 to 2.82) (Figure 3(c)).

**Figure 4:**
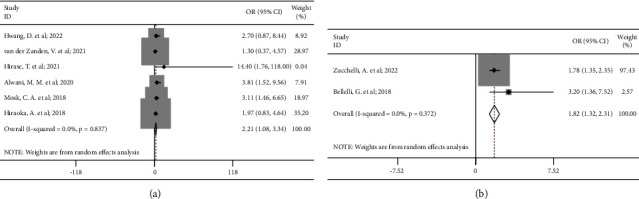
Subgroup analyses of the associations between low skeletal muscle mass and the incidence of delirium in (a) patients undergoing major surgeries; (b) patients without surgeries.

**Figure 5:**
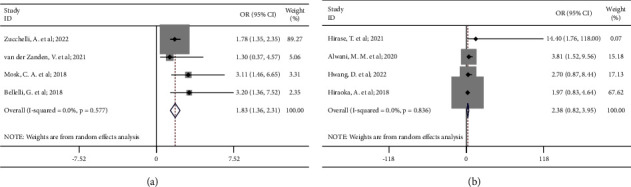
Subgroup analyses of the associations between low skeletal muscle mass and the incidence of delirium in (a) patients ≥75 years old; (b) patients <75 years old.

In subgroup analysis, we observed significant associations between low skeletal muscle mass and incidence of delirium in both patients with or without major surgeries, but low skeletal muscle mass was associated with a higher incidence of delirium in patients undergoing major surgeries (OR 2.21, 95% CI 1.08 to 3.34; *P*_heterogeneity_=0.84; Figure 4(a)) than those without surgeries (OR 1.82, 95% CI 1.32 to 2.31; *P*_heterogeneity_=0.37; Figure 4(b). Furthermore, subgroup analysis of age indicated that low skeletal muscle mass was significantly associated with an increased incidence of delirium in patients ≥75 years old (OR 1.83, 95% CI 1.36 to 2.31; *P*_heterogeneity_=0.58; Figure 5(a)) instead of those <75 years old (OR 2.38, 95% CI 0.82 to 3.95; *P*_heterogeneity_=0.84; Figure 5(b)).

## 4. Discussion

To our knowledge, this study is the first systematic review and meta-analysis investigating the associations between low skeletal muscle mass and the incidence of delirium in hospitalized patients. The initial meta-analysis of the whole nine studies did not yield a significant association between low skeletal muscle mass and the incidence of delirium. Therefore, we performed a sensitivity analysis and removed the study by Pernik et al. [15] which could increase the risk of bias. The subsequent analyses indicated that low skeletal muscle mass was significantly associated with an increased incidence of delirium in hospitalized patients, particularly in those undergoing major surgeries or ≥75 years old. Among the studies included in this systematic review and meta-analysis, three studies reported no significant association between low skeletal muscle mass and the incidence of delirium [13, 24, 26]. The reasons we speculated might include younger age and lower incidence of delirium. For example, participants in the two studies reporting nonsignificant association had a mean age of <75 years old [13, 26], while other participants in the studies of significant association reported by Zucchelli et al. for instance, had a mean age of over 80 years old [16, 27]. Furthermore, in another study reporting nonsignificant association [24], the incidence of postoperative delirium is just about 5.7% and 4.4% in ovarian cancer patients with low and normal muscle mass, respectively, and there were only 11 patients having postoperative delirium. Therefore, it is hard to get a significant result from this study with such a low incidence of postoperative delirium. Considering the controversial results among the included studies, more studies with large sample are required in the future.

Skeletal muscle is one of the largest metabolic organs of the human body, and it is also an important endocrine organ that secretes myokines in autocrine, paracrine, and endocrine manners for communicating with other systems [32, 33]. The relationship between skeletal muscle and brain function has attracted more and more attention [34]. It was reported that several myokines such as cathepsin B and interleukin-6 can cross the blood-brain barrier and directly regulate neurotrophic signaling in brain regions relevant for cognitive functions (hippocampus and prefrontal cortex) and metabolic regulation (hypothalamus). Some myokines can also stimulate the secretion of neurotrophins including brain-derived neurotrophic factor (BDNF) and fibronectin type III domains containing protein 5 (FNDC5), which could improve cognitive function [33, 35]. Furthermore, a recent animal study reported that in a preoperative muscle atrophy model induced by tail suspension, rats with low skeletal muscle mass were associated with an increased occurrence of the perioperative neurocognitive disorder, and a reduced level of BDNF was also observed [36]. Besides basic studies, a recent meta-analysis also suggested that sarcopenia was associated with an increased risk of cognitive impairment [37]. Therefore, the cross-talk between skeletal muscle and brain might explain why low skeletal muscle mass is associated with an increased incidence of delirium.

Low skeletal muscle mass and delirium also have several common risk factors including increased age, malnutrition, and severe illness [1, 3, 8, 9]. In particular, age is recognized as a primary cause of sarcopenia, and the skeletal muscle mass can be lost rapidly after the age of 75 years old [1, 3]. It may explain why our subgroup analyses found that low skeletal muscle mass was significantly associated with an increased incidence of delirium only in patients ≥75 years old instead of those <75 years old. Furthermore, the incidence of postoperative delirium is relatively high in patients undergoing major surgeries, because surgery procedures, infection, general anesthesia, pain, acute stress response, and systemic inflammation are common risk factors for postoperative delirium [10, 38, 39]. Our previous study indicated that sarcopenia could increase the risk of multiple postoperative complications [7], which might promote the postoperative stress response and systemic inflammation. Therefore, low skeletal muscle mass could lead to a higher incidence of delirium in patients undergoing major surgeries than those without surgeries as we found in the subgroup analyses.

Considering the associations between skeletal muscle and delirium, the ways of improving skeletal muscle mass might be used for preventing delirium. For age-related sarcopenia, only physical exercise instead of nutritional intervention alone or specific drugs is recommended as the primary treatment of skeletal muscle loss [1]. Furthermore, some studies have also shown the benefits of physical exercise in reducing the incidence of delirium in hospitalized patients [40–42]. Therefore, physical exercise and skeletal muscle mass promotion could be important for the prevention and management of delirium in hospitalized older patients.

The strengths of this study were that we first conducted this systematic review and meta-analysis about the associations between low skeletal muscle mass and incidence of delirium. Through the main meta-analysis and subgroup analyses, we found that low skeletal muscle mass was significantly associated with an increased incidence of delirium in hospitalized patients, particularly in those undergoing major surgeries or ≥75 years old. Our results could be helpful to identify the hospitalized patients with a high risk of delirium.

There were also several limitations of this study. First, the number of included studies was relatively small, and we found a significant existence of publication bias. To compensate for this limitation, we conducted a trim-and-fill analysis, and the adjusted pooled result remained constant. Second, the included studies did not have a consistent assessment and definition of low skeletal muscle mass and delirium, which could increase the risk of bias. However, limited by the small number of included studies, subgroup analyses about this problem were hard to conduct, and further studies with a large sample and consistent assessment of this issue are required in the future. Third, some studies indicated that a higher BMI could be associated with an increased risk of postoperative delirium [29–31], indicating that BMI might be a confounding factor. However, several studies in this systematic review and meta-analysis did not report the BMI of the participants, and the other studies have reported BMI in inconsistent ways (mean, median or categorical data). It is hard for us to describe the BMI of included participants or conducting subgroup analyses about the BMI, therefore, we call for studies to focus on this issue in the future.

In conclusion, low skeletal muscle mass is associated with an increased incidence of delirium in hospitalized patients, particularly in those of older age and undergoing major surgeries. Therefore, great attention should be paid to these patients. Considering the limitations of this systematic review and meta-analysis, further prospective observational studies about the associations between skeletal muscle mass and delirium are required in the future.

## Figures and Tables

**Table 1:** Characteristics of included studies.

Author; year	Country	Study design	Assessment of skeletal muscle mass	Definition of low skeletal muscle mass	Assessment of delirium	Study population	No. of patients (*n*)	NOS quality assessment
Pernik et al. 2022 [15]	America	Retrospective cohort study	PMI (by CT and MRI)	PMI quartile 4	A chart review method	Patients ≥65 years old receiving thoracolumbar spine surgery	196	7
Hwang et al. 2022 [13]	South Korea	Retrospective cohort study	Appendicular SMI (by BIA)	SMI (males, <7.0 kg/m^2^; females, <5.7 kg/m^2^)	Based on the diagnostic and statistical manual of mental disorders, 4th edition	Patients with a mean age of over 70 years old receiving total knee arthroplasty	452	8
Zucchelli et al. 2022 [16]	Italy	Cross-sectional study	Based on the calf circumference	Calf circumference ≤34 cm for males, ≤33 cm for females	Based on a 4AT score	Patients with a mean age of 83.1 years old and were admitted to acute hospital medical wards, emergency departments, rehabilitation wards, nursing homes, and hospices	1675	8
van der Zanden et al. 2021 [24]	The Netherlands	Retrospective cohort study	SMI at the L3 level (by CT)	SMI <38.50 cm^2^/m^2^	Not reported	Women with a median age of 75.9 (from 70 to 89) years old receiving surgery for ovarian cancer	213	7
Hirase et al. 2021 [25]	America	Retrospective cohort study	PMI (by CT and MRI)	PMI below the gender specific cut-off values determined using receiver operating characteristic curves and youden index	Not reported	Patients with a mean age of 60.1 years old receiving complex revision thoracolumbar spine surgery	114	7
Jones et al. 2020 [28]	America	Case-control study	SMI at the L3 level (by CT)	SMI ≤41.6 cm^2^/m^2^ for males and ≤32.0 cm^2^/m^2^ for females	Not reported	Patients with a mean age of 59.4 years old receiving autologous free tissue reconstruction following resection of head and neck malignancy	168	6
Mosk et al. 2018 [14]	The Netherlands	Retrospective cohort study	SMI at the L3 level (by CT)	Patients in the lowest sex-specific quartile for skeletal muscle mass	Based on the delirium observational screening scale	Patients with a median age of 76 (from 73 to 80) years old receiving elective surgery for colorectal cancer	251	8
Bellelli et al. 2018 [27]	Italy	Cross-sectional study	Whole body SMI (by BIA)	SMI of less than 8.87 and 6.42 kg/m^2^ in men and women, respectively	Based on the short portable mental status questionnaire	Patients with a mean age of 80.9 years old and were admitted to the hospital wards	588	8
Hiraoka et al. 2018 [26]	Japan	Retrospective cohort study	PMI (by CT)	PMI ≤4.24 cm^2^/m^2^ for males, ≤2.50 cm^2^/m^2^ for females	Based on the intensive care delirium screening checklist	Patients with a mean age of 70.4 years old receiving surgical resection for hepatocellular carcinoma	171	8

BIA, bioimpedance analysis; CT, computed tomography; MRI, magnetic resonance imaging; NOS, newcastle-ottawa-scale; PMI, psoas muscle index; SMI, skeletal muscle index.

## Data Availability

All data generated or analyzed during this study are included in this published article.

## Supplementary Materials

Supplementary Materials Supplementary material 1: Search strategies for each database. Supplementary material 2: PRISMA_2020_checklist. Supplementary material 3: MOOSE checklist for meta-analyses of observational studies.
